# Ginsenoside Rb1 attenuates neuroflammation via activating Wnt/β-catenin signaling pathway to exert neuroprotective effect on cerebral ischemic-reperfusion injury

**DOI:** 10.3389/fnagi.2025.1555067

**Published:** 2025-08-06

**Authors:** Ruo-jing Liu, Xue Zhao, Yi-zhen Zhu, Ling-Ling Fu, Guo Ge, Jun-de Zhu

**Affiliations:** ^1^Department of Human Anatomy, Guizhou Medical University School of Basic Medicine, Guizhou, China; ^2^Key Laboratory of Brain Function and Diseases Tissue Bank of Higher Education Institutions in Guizhou Province, Guizhou, China; ^3^Department of Radiology, Guiqian International General Hospital, Guizhou, China; ^4^Class 5, Grade 2023, Guizhou Medical University, Guizhou, China; ^5^Key Laboratory of Molecular Biology, Guizhou Medical University, Guizhou, China

**Keywords:** ginsenoside Rb1, Wnt/β-catenin signaling pathway, microglia polarization, neuroinflammation, neuroprotection

## Abstract

**Purpose:**

To explore the molecular mechanism of G-Rb1 regulating microglia polarization through Wnt/β-catenin signaling pathway to alleviate cerebral ischemia-reperfusion injury in mice.

**Methods:**

C57BL/6J mouse middle cerebral artery occlusion/reperfusion (MCAO/R) model and microglia (BV2) oxygen-glucose deprivation/reoxygenation (ODG/R) model were used. The neuroprotective effect of G-Rb1 *in vivo* and *in vitro* was evaluated by measuring nerve function deficit, cerebral blood perfusion recovery, infarct volume and cell viability. Immunofluorescence, flow cytometry, Western blot and qRT-PCR were used to evaluate the effects of G-Rb1 on the Wnt/β-catenin signaling pathway and microglia phenotypic polarization mediated neuroinflammation *in vivo* and *in vitro*.

**Results:**

Compared with the Sham group, the symptoms of neurological impairment, cerebral blood perfusion, cerebral infarction volume and inflammatory reaction were increased in the IRI group. Compared with the IRI group, G-Rb1 group showed less symptoms of neurological impairment, increased cerebral blood perfusion, decreased cerebral infarction volume, increased proportion of M2-type microglia, increased release of anti-inflammatory factors, reduced inflammatory response, and up-regulated β-catenin expression while down-regulated GSK-3β expression. It was demonstrated that G-Rb1 activates the Wnt/β-catenin signaling pathway after CIRI. Compared with G-Rb1 group, G-Rb1 + XAV939 group had more neurological impairment, increased cerebral infarction volume, increased M1 microglia proportion, and increased neuroinflammation. Meanwhile, β-catenin expression decreased while GSK-3β expression increased. The results of *in vitro* experiments were similar to those of *in vivo*, which demonstrated that G-Rb1 may alter microglial polarization phenotype through Wnt/β-catenin signaling pathway and alleviate neuroinflammatory response after CIRI.

## Introduction

Stroke has a high incidence, significant disability rate, and elevated mortality. Approximately 790,000 individuals experience new or recurrent stroke annually, with ischemic stroke accounting for 85% of cases (Tsao et al., 2022). The primary causes of ischemic stroke are cerebral artery stenosis or embolism, resulting in the interruption of blood supply and subsequent cerebral tissue ischemia, hypoxia, and necrosis (Mendelson and Prabhakaran, 2021). Currently, the main clinical treatment approaches for ischemic stroke both domestically and internationally involve intravenous administration of recombinant tissue-type plasminogen activator (t-PA) and intravascular mechanical thrombectomy (Powers et al., 2019; Herpich and Rincon, 2020), aiming to promptly restore hemoperfusion. However, these two treatments have stringent time window requirements. t-PA necessitates initiation within 4–5 h following the onset of stroke (Majidi et al., 2019), while mechanical thrombectomy’s time window is approximately 6 h (Mozaffarian et al., 2016). Consequently, only a small proportion of patients (1–2%) can benefit from these interventions. Clinically speaking, most vascular reperfusion is deemed “ineffective reperfusion,” primarily due to Cerebral Ischemia/Reperfusion Injury (CIRI) (Stoll and Nieswandt, 2019; Ding et al., 2020). CIRI is considered one of the most crucial complications after an ischemic stroke; its pathogenesis predominantly involves oxidative stress, neurotoxic effects induced by excitatory amino acids, intracellular calcium overload autophagy as well as inflammation processes (Zhang Q. et al., 2022). Inflammation plays a pivotal role throughout the course of CIRI.

Microglia are essential components of the innate immune surveillance network in the central nervous system. In the resting state, microglia are highly dynamic cells, enabling them to rapidly respond to inflammation by transitioning into an activated state. Alterations in microglial phenotype and function can be observed across various neuropathological conditions. Following cerebral ischemia, microglia are the first responders to neural tissue injury, and their activation constitutes a critical event in the post-trauma cascade. Extensive research has demonstrated that microglia exhibit multiple distinct activation states upon stimulation. Currently, it is widely accepted that microglial phenotypes can be categorized into classical activation (M1), alternative activation (M2a), type II alternative activation (M2b), and acquired deactivation (M2c) (Malyshev and Malyshev, 2015). The M1 phenotype predominantly exerts neurotoxic effects by producing pro-inflammatory cytokines and forming glial scars. In contrast, the M2a phenotype is primarily associated with phagocytosis, clearing cellular debris and promoting tissue regeneration. The M2b phenotype enhances the release of anti-inflammatory factors via immune regulation (Walker and Lue, 2015). However, numerous studies have indicated that activated microglia do not exclusively adopt a single phenotype. Instead, during various stages of spinal cord injury, both M1 and M2 microglia coexist at the injury site (Akhmetzyanova et al., 2019). The M1/M2 paradigm represents a simplified model reflecting two opposing roles in the inflammatory response: when the proportion of M1 microglia predominates, the body exhibits pro-inflammatory effects; conversely, when M2 microglia dominate, anti-inflammatory and neuroprotective effects are observed. It has been reported that during the early stages of cerebral ischemia, the periinfarction area is primarily activated by M2 microglia; however, after a prolonged period following blood perfusion restoration, M1 microglial activation becomes predominant (Luo et al., 2022), thereby aggravating CIRI. Activated microglia and apoptotic neurons are predominantly found within the peri-infarction area; hence the regulation of microglia within this region serves as a key measure for neuronal protection.

The Wnt/β-catenin signaling pathway is involved in physiological and pathological processes such as cell proliferation, differentiation, neurogenesis and inflammatory response (Wang et al., 2013). The pathway exerts its function by regulating the activation of the transcriptional coactivator β-catenin and subsequently controlling downstream gene expression (Harmon et al., 2016). Studies suggest that activation of the Wnt/β-catenin signaling pathway can facilitate neuroinflammatory recovery and angiogenesis following CIRI (Liu et al., 2022), while stimulating this pathway can induce neuronal differentiation and improve the inflammatory microenvironment following cerebral ischemic injury (Song et al., 2019). In hemorrhagic stroke models, TWS119, an agonist of the Wnt/β-catenin signaling pathway, inhibits proinflammatory cytokine release and promotes a shift in microglial phenotype from M1 to M2 phenotype. XAV939, an inhibitor targeting the Wnt signaling pathway (Huang et al., 2009), which specifically inhibits β-catenin transcription (Wang et al., 2022), was used to block the activation of the pathway and observe whether there was any effect of G-Rb1 through the Wnt/β-catenin signaling pathway.

Ginsenosides are primarily classified into protopanaxadiols, protopanaxatriols, and oleanane, with protopanaxadiols exhibiting the highest activity, including Rb1, Rb2, Rc, Rd, Rg3, Rh2 (Park et al., 2022). Among these components, Ginsenoside Rb1 (G-Rb1) is considered one of the most potent constituents in ginseng. Recent studies have revealed that G-Rb1 possesses diverse biological activities such as antioxidant properties and anti-inflammatory and anti-apoptotic effects, suggesting its potential therapeutic benefits for neurodegenerative diseases (Ahmed et al., 2016; Manju and Bharadvaja, 2024). Numerous investigations have explored the therapeutic effects of G-Rb1 on both *in vivo* and *in vitro* central nervous system models (Xie et al., 2021). For instance, G-Rb1 demonstrates remarkable antioxidant and anti-apoptotic effects during cerebral ischemia by inducing SOD-1 expression (Kim et al., 2022). Furthermore, G-Rb1 mitigates cortical neuron damage by downregulating superoxide production and TNF-α expression in hypoxia-activated microglia (Ke et al., 2014). However, whether G-Rb1 influences the microglia polarization following hypoxia activation or if it involves the Wnt/β-catenin signaling pathway in its neuroprotective effect remains unclear.

Therefore, we investigated the neuroprotective effect of G-Rb1 against CIRI mediated via the Wnt/β-catenin signaling pathway in the study. These results offer a novel therapeutic target for clinical management of CIRI and provide further experimental evidence supporting the clinical application of traditional Chinese medicine.

## Materials and methods

### Animals and groups

Adult (8–10 week, weighing 20–25 g) male C57BL/6J mice provided by the Experimental Animal Center of Guizhou Medical University (license number: SYXK (Gui) 2023–0002). The experiment was approved by the Animal Ethics Committee of Guizhou Medical University (approval number: 2200094). The mice were allowed to eat and drink freely and were housed under a 12-h light/dark cycle.

Mice were randomly into four groups: sham operation group (Sham), ischemic reperfusion injury group (IRI), Ginsenoside Rb1 group (G-Rb1) and Ginsenoside Rb1 + XAV939 group (G-Rb1 + XAV939) (*n* = 25/group).

Except the Sham group, all groups were operated for middle cerebral artery occlusion/reperfusion (MCAO/R) to induce cerebral ischemic-reperfusion model. IRI group: mice injected intraperitoneally with normal saline every day for 3 days before MCAO/R modeling. G-Rb1 group: mice were intraperitoneally injected with G-Rb1 (40 mg/kg) (周璐 et al., 2023) every day for 3 days before MCAO/R modeling, and the dose has been proved to have neuroprotective effect in our previous study. G-Rb1 + XAV939 group: mice were intraperitoneally injected with G-Rb1 and XAV939 (5 mg/kg) (Zhao et al., 2020) every day for 3 days before MCAO/R modeling. The mice were sacrificed on day 1, 3, 7, 14 after surgery.

### MCAO/R modeling

A modified Zea-Longa method was used to establish an MCAO/R model (Longa et al., 1989). pentobarbital sodium (50 mg/kg) was used to anesthetize mice by intraperitoneal injection. The muscles were dissected from the edge of the sternocleidomastoid muscle. the common carotid artery (CCA), internal carotid artery (ICA), and external carotid artery (ECA) were dissected. Firstly, the proximal end of the CCA and the distal end of the ECA were ligated. Secondly, the ICA was temporarily clipped with an artery clamp. Thirdly, we cut a small hole from the ECA bifurcation, inserted a thread plug into the ICA, gently pushed the thread plug using tweezers, and tightened the end of the line when the insertion depth was about 1.2 cm from ECA. 50 min after the induction of ischemic, the thread plug was pulled out and the distal end of the ECA was immediately tightly fastened to prevent bleeding. Then, the ligature was released from the proximal of the CCA, and blood was returned to the brain. The incision was sutured and disinfected, and the temperature of the mice was monitored in real time until they woke up.

### Drugs

Ginsenoside Rb1 (HPLC>98%, yuanyebio, catalog MFCD00153848, China), Wnt/β-catenin signaling pathway inhibitor XAV939 (APExBIO, catalog A1877, China), LPS (Sigma-Aldrich, catalog L2630, Germany), 2% triphenyltetrazolium chloride (TTC) dye solution (Beijing Solarbio, catalog G3005, China), MCAO/R model thread bolt (Beijing Xinong, catalog A4-162350, China), GSK-3β rabbit polyclonal primary antibody (catalog WL01456), β-catenin rabbit polyclonal primary antibody (catalog WL03554), TNF-α rabbit polyclonal primary antibody (catalog WL01581), IL-1β rabbit polyclonal primary antibody (catalog WLH3903) were obtained from Wanleibio. Iba1 rabbit polyclonal primary antibody (catalog ab178847) and Arg-1 mouse polyclonal primary antibody (catalog ab239731) were obtained Abcam, iNOS mouse polyclonal primary antibody (Thermo Fisher Scientific, catalog MA5-17139, United States), HRP-labeled rabbit antibody (Wuhan Sanying Biotech, catalog SA00001-2, China), Cell Counting Kit-8(APExBIO, catalog K1018, China), BCA assay kit (Wanleibio, catalog WLA004, China) confocal laser microscopy (Olympus, Japan), RNA extraction kit and reverse transcriptase kit (Thermo Fisher Scientific, catalog AM9775 and K1691, United States), laser speckle blood flow imaging system (RWD, catalog RFLSI ZW, China).

### Cell culture and OGD/R modeling

Mouse microglia cell line (BV2, CL-0493) was purchased from Procell Life Science & Technology (Wuhan, China). Cells were divided into the following groups: (1) Control group: cells were cultured in normal culture medium without OGD/R. (2) LPS group: cells were treated with LPS (100 ng/mL) for 6 h prior to OGD and subsequently subjected to OGD for 6 h. Then the OGD medium was replaced with the normal medium and the cells incubated for 24 h in normal condition. (3) G-Rb1 group: cells were treated with LPS and G-Rb1 (10 μM) for 6 h before OGD, then the same OGD/R model was subjected. (4) G-Rb1 + XAV939 group: cells were treated with LPS, G-Rb1 and inhibitor XAV939 (30μM) (Zhang Y. N. et al., 2022) for 6 h before OGD, then the same OGD/R model was subjected.

### Cell viability assay

Cell viability was detected with the Cell Counting Kit-8(CCK-8), After OGD/R treatment, cell (1 × 10^4^ per well) were inoculated in 96-well plates with normal medium and 10μL CCK-8 solutions were added in each well for 3 h at incubator. Finally, the cell viability will determine by spectrometry at 450 nm after incubation.

### Behavior test

At 1, 3, 7, 14 days post-reperfusion, the Zea-Longa (Longa et al., 1989) scoring method and mNSS scoring method (Chen et al., 2001) were used to evaluate the neurological function deficits in mice. The scoring criteria of the Zea-Longa scoring method were as follows: 0 score: no symptoms of nerve injury, 1 point: cannot fully extend the contralateral front claw, 2 points: rear-end turn to the opposite side, 3 points: slouching to the opposite side, 4 points: unable to walk spontaneously, loss of consciousness. Mice that did not meet qualification standards were excluded, and subsequent experiments were conducted within the same batch to ensure equal group sizes. The mNSS is a composite test of motor, reflexes, sensory system and balance, and the higher score indicate more severe neurological impairment. The scoring criteria of the mNSS scoring were as follows: A score of 13–18 indicates severe injury, 7–12 indicates moderate injury, and 1–6 indicates mild injury. 10 mice were excluded due to criteria limitations set for mNSS scoring system in our experiments.

### Laser speckle blood flowmeter measurement

Mice were anesthetized with pentobarbital sodium (50 mg/kg) at 1, 3, 7, 14 days post-reperfusion, followed by routine skinning and disinfection. A longitudinal incision was made between both ears and eyes of the mice, followed by removal of the surface fascia using tweezers. Subsequently, the exposed skull was secured with a retractor. The monitoring area for both left and right hemispheres of the mice was carefully selected to be identical, enabling detection of blood perfusion information using laser speckle blood flow system. Cerebral blood flow (CBF) (%) = (affected side/normal side) × 100%.

### TTC staining

Mice were sacrificed at 1, 3, 7, 14 days post-reperfusion, and frozen brains were slice into coronal sections of 2 mm thickness. The slices were stained with 2% TTC solution at 37°C and incubated in darkness for 20 min. Following fixation with a 4% Paraformaldehyde (PFA) solution for an extended period of 24 h, the infarct volume was visually examined and documented. Utilizing Image J software, analysis was conducted to determine the infarct area within each slice and calculate the corresponding infarct volume ratio.

### Immunofluorescence

Mice were sacrificed at 1, 3, 7, 14 days post-reperfusion, rapid perfusion was performed using 100 mL of normal saline and 60 mL of 4% PFA solution. The brains were fixed for 24 h in 4% PFA at room temperature. After dehydration and clearing, the brains were embedded in paraffin and the samples were coronally sectioned at 5 μM thickness. Prior to staining, the sections were rinsed 3 times with 0.01M PBS for 5 min followed by immunofluorescence staining using polyclonal primary antibodies against Iba1, iNOS, Arg-1 (1:200) overnight at 4°C prior to incubation with corresponding fluorescent secondary antibodies along with DAPI (1:500) at room temperature for 2 h. The procedure of cell immunofluorescence staining was consistent with that of tissue. Laser confocal microscopy was used to obverse and photograph tissue and cell sections, and then Image J was used to count positive cells.

### Western blot

Total protein of the brain tissue and BV2 cells was extracted and quantified by using a BCA assay kit, electrophoresis was performed at 120 V for 80–120 min, followed by transfer to nitrocellulose membrane at 400 mA. 5% skim milk powder closed for 2 h, and then incubated with primary antibody, mouse polyclonal Anti-Arg-1, rabbit polyclonal Anti-CD206, rabbit polyclonal Anti-TNF-α, rabbit polyclonal Anti-IL-1β (1:1,000) overnight 4°C. Immunodetection was performed by electrochemiluminescence after incubation with horseradish peroxidase goat anti-rabbit antibody (1:5,000). Gray value of protein bands was determined and analyzed by Image J.

### Quantitative real-time polymerase chain reaction

Total RNA was extracted from the cerebral parietal cortex by using the RNA extraction kit, the concentration and purity of RNA were measured, and reverse transcription reaction was performed using the rapid reverse transcription kit. Data analysis was performed using GAPDH as the baseline and the 2 ^–ΔΔ^*^ct^* value analysis. The sequence of primers used in this study is shown in Table 1.

**TABLE 1:** Primers sequence.

Primers	Forward (5′–3′)	Reverse (5′–3′)
IL-1β	TGTCTTGGCCGAGGACT AAGG	TGGGCTGGACTGTTTCT AATGC
TNF-α	GACGTGGAACTGGCAG AAGAG	TTGGTGGTTTGTGAGTG TGAG
Arg-1	TCACCTGAGCTTTGAT GTCG	CTGAAAGGAGCCCTGTC TTG
CD206	CAAGGAAGGTTGGCATT TGT	CCTTTCAGTCCTTTGCA AGC
GAPDH	AGACAGCCGCATCTTCT TGT	TACTCAGCACCAGCATC ACC

IL-1β, interleukin-1β. TNF-α, Tumor Necrosis Factor-α. Arg-1, arginase-1. CD206, Mannose receptor. GAPDH, glyceraldehyde-3-phosphate dehydrogenase.

### Statistical analyses

GraphPad Prism 8.0 statistical software was used for data analysis and graphing. All data are expressed as mean ± standard deviation(*−x* ± *s*). For data from three groups or more, one-way ANOVA was first used, followed by *t*-test between groups. The two groups of data were compared using independent sample *t*-test or two-factor analysis of variance. When the data are not normally distributed or the differences between groups are large, non-parametric tests (such as Kruskal-Wallis test or Mann-Whitney U test) are used, and *P* < 0.05 is considered to be significant.

## Results

### G-Rb1 promote the transformation of microglia into M2 type and exert anti-inflammatory effects *in vitro*

**FIGURE 1:**
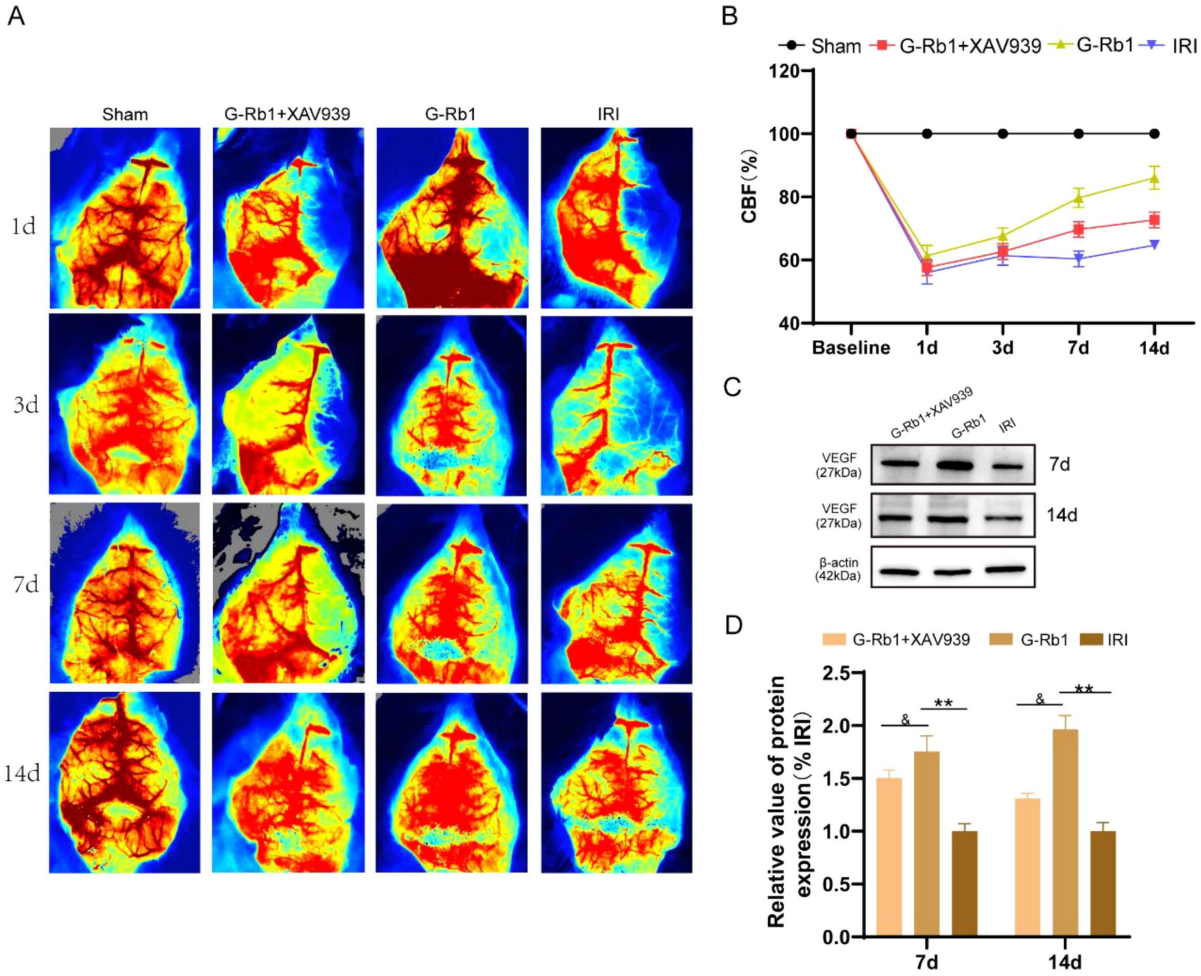
G-Rb1 exhibit increased cerebral blood flow and vascular density in MCAO/R mice. **(A)** Laser speckle flow results at 1, 3, 7, 14 days post-reperfusion of the mice (*n* = 3). **(B)** Statistical analyses of the CBF at 1, 3, 7, 14 days post-reperfusion of the mice (*n* = 3). **(C,D)** Protein expression of VEGF at 7 and day 14 post-reperfusion of the mice (*n* = 3).^**^P < 0.01 vs. the IRI group, ^&^*P* < 0.05 vs. the G-Rb1 group.

After treatment with G-Rb1 of different concentrations for 3, 6, 12, 24 h, CCK-8 was used to detect cell viability. And the results showed that BV2 cells treated with G-Rb1 (10 μM) showed advantages at various time points (Figure 1A), especially at 6 h showed the best cell viability, while at 12 and 24 h the cell viability decreased significantly. Moreover, when the concentration of G-Rb1 was greater than 15 μM, the cell viability of BV2 cells could significantly decrease in both dose- and time-dependent manner. Therefore, the action concentration and time of G-Rb1 were determined to be 10 μM and 6 h *in vitro*.

Twenty four hours following OGD/R modeling, immunofluorescence and Western bolt were used to detect the phenotypic polarization of BV2 cells and the expression of inflammatory factors. The results showed that iNOS fluorescence intensity of BV2 cells in the LPS group was significantly enhanced compared with the Control group. And compared with the LPS group, iNOS fluorescence intensity of BV2 cells in the G-Rb1 group decreased while the fluorescence intensity of Arg-1 increased, indicating that G-Rb1 promoted the anti-inflammatory M2 polarization of BV2 cells. The G-Rb1 + XAV939 group showed an increase in iNOS fluorescence intensity and a decrease in Arg-1 fluorescence intensity compared with the G-Rb1 group (Figures 1B), which proved that after the addition of inhibitor XAV939, G-Rb1 reduced the protective effect of BV2 cells to the M2 phenotype. Western blot results were consistent with immunofluorescence results: compared with the Control group, iNOS protein expression in the LPS group was increased while Arg-1 protein expression was decreased (Figures 1C,D, *P* < 0.01). Compared with the LPS group, the protein expression of Arg-1 in the G-Rb1 group was significantly increased (*P* < 0.01) while the protein expression of iNOS was decreased (Figures 1C,D, *P* < 0.01). And compared with the G-Rb1 group, iNOS expression in the G-Rb1 + XAV939 group increased (*P* < 0.01) while Arg-1 expression decreased (Figures 1C,D, *P* < 0.01), indicating that G-Rb1 can reduce the release of pro-inflammatory factors and increase the expression of anti-inflammatory factors, and the effect is weakened after adding XAV939.

### Effect of G-Rb1 on the recovery of neurological function in cerebral ischemic mice

**FIGURE 2:**
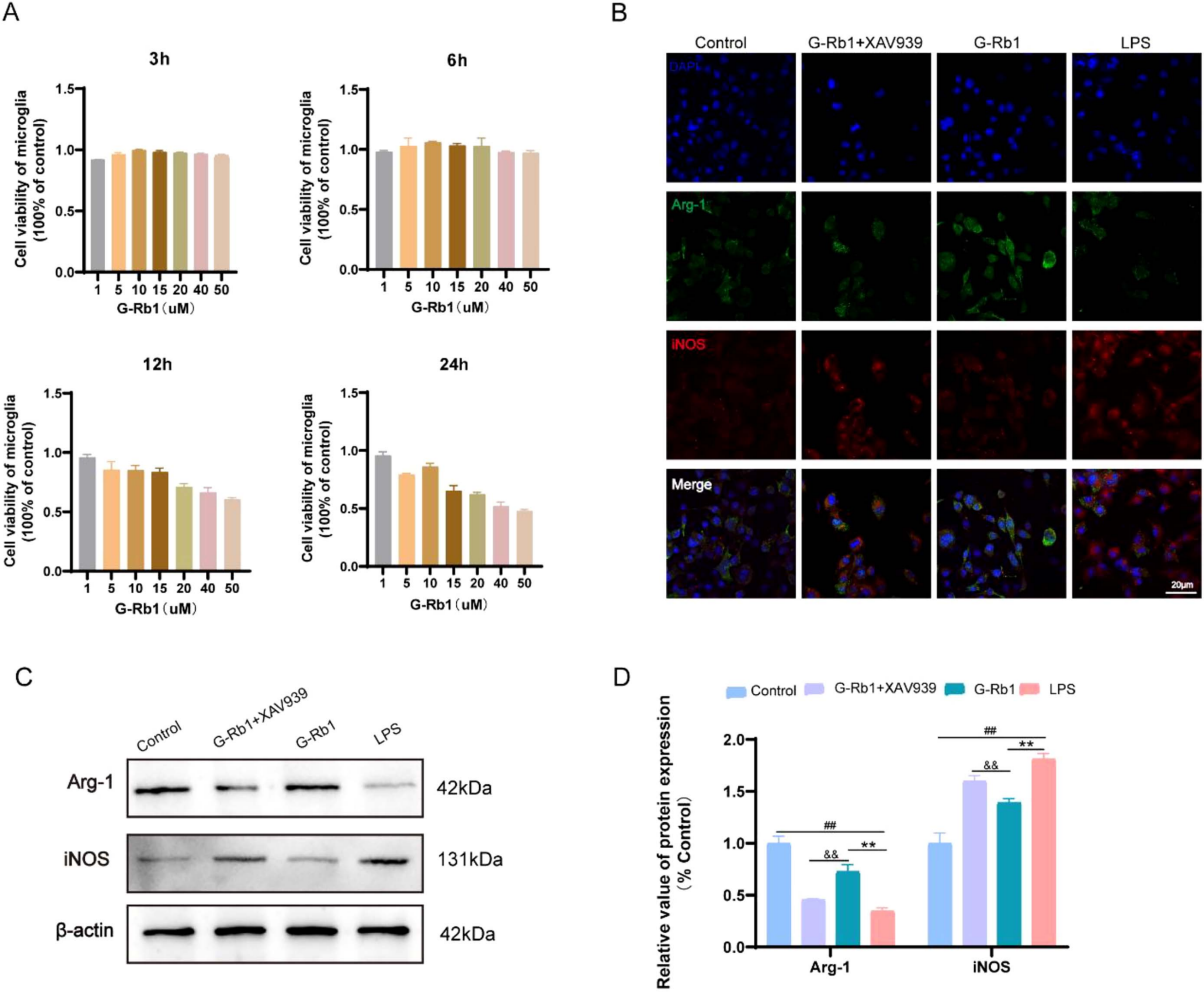
G-Rb1 promote the transformation of microglia into M2 type and exert anti-inflammatory effects *in vitro*. **(A)** Cell viability of BV2 cells treated with different concentrations of G-Rb1 (*n* = 5). **(B)** Representative immunofluorescence images showing the expression of iNOS and Arg-1 in BV2 cells (*n* = 3) (bar = 20 μm). **(C,D)** rotein expression of iNOS and Arg-1 of BV2 cells (*n* = 3). ^##^*P* < 0.01 vs. the Sham group, ^**^P < 0.01 vs. the IRI group, ^&⁣&^*P* < 0.01 vs. the G-Rb1 group.

To assess the neuroprotective effects of G-Rb1, we evaluated neurological deficits and infarct volume at 1, 3, 7, 14 days post-reperfusion of the mice (Figure 2). Results showed that the IRI group deteriorated neurological function deficit (Figures 2A,B, *P* < 0.05) and a larger infarct volume (Figures 2C,D, *P* < 0.01) compared with the Sham group, with the most severe deficits and infarct volume observed at 3th day, followed by a gradual recovery. The G-Rb1 group significantly reduced neurological deficits (Figures 2A,B, *P* < 0.05) and infarct volume (Figures 2C,D, *P* < 0.05), particularly at 7th day and 14th day post-reperfusion compared with the IRI group, suggesting that G-Rb1 mainly exerts neuroprotective effects during the recovery period following cerebral ischemia-reperfusion injury. Results also showed that the G-Rb1 + XAV939 group deteriorated neurological function (Figures 2A,B, *P* < 0.05) and a severer infarct volume (Figures 2C,D, *P* < 0.05) compared with the G-Rb1 group at 1, 3, 7, 14 days post-reperfusion of the mice. These results suggest that XAV939 counteracted the neuroprotective effect of G-Rb1, and aggravated the neurological deficits and infarct volume following cerebral ischemia-reperfusion injury.

### G-Rb1 exhibit increased cerebral blood flow and vascular density in MCAO/R mice

**FIGURE 3:**
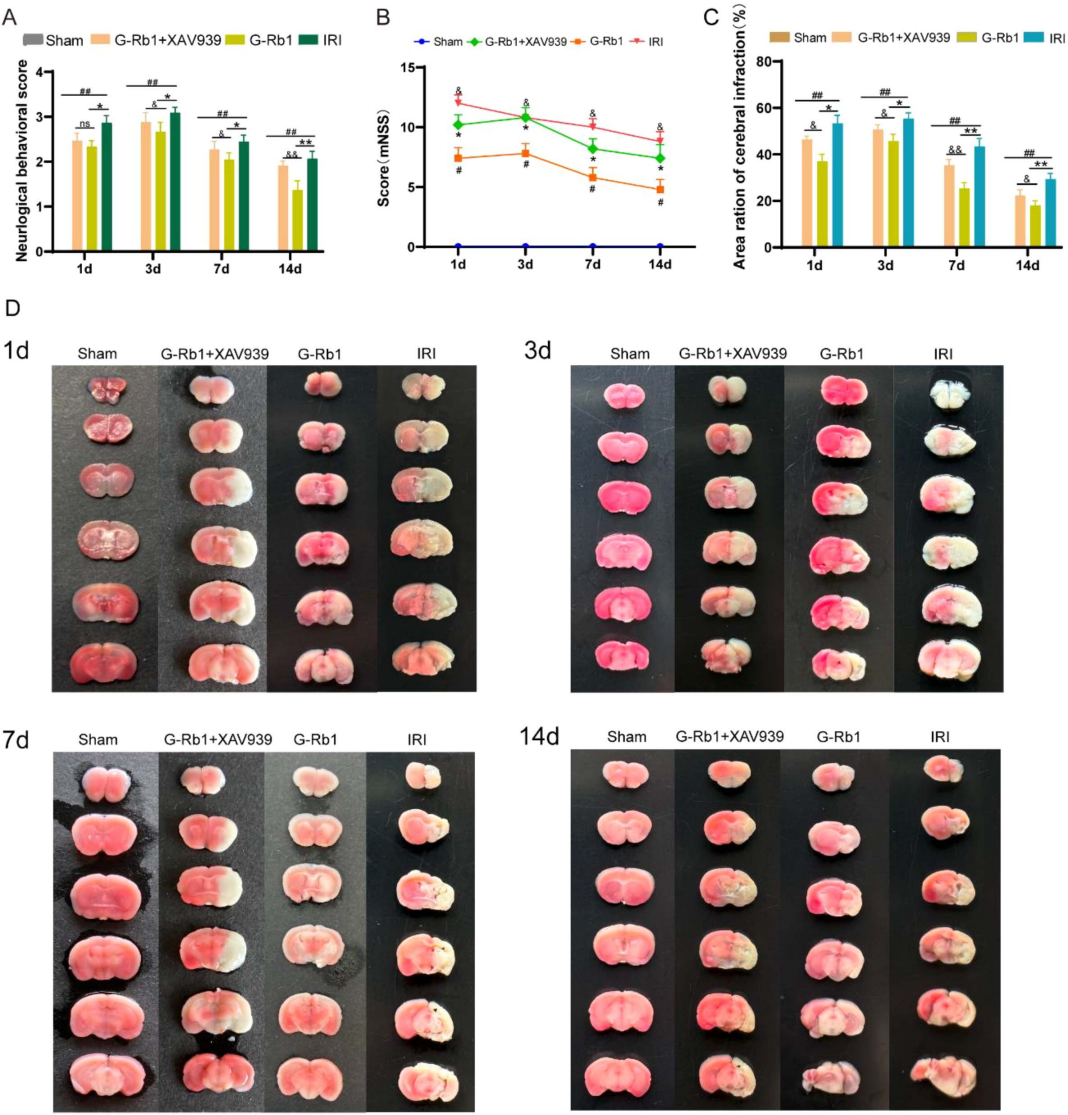
Effect of G-Rb1 on the recovery of neurological function in cerebral ischemic mice. **(A)** Statistical analyses of the Zae-Longa score at 1, 3, 7, 14 days post-reperfusion of the mice (*n* = 10). **(B)** Statistical analyses of the mNSS score at 1, 3, 7, 14 days post-reperfusion of the mice (*n* = 10). **(C)** Statistical analyses of brain infarct volume at 1, 3, 7, 14 days post-reperfusion of the mice (*n* = 3). **(D)** TTC staining results of the mice at 1, 3, 7, 14 days post-reperfusion of the mice. ^#^*P* < 0.05, ^##^*P* < 0.01 vs. the Sham group, *P < 0.05, ^**^P < 0.01 vs. the IRI group, ^&^*P* < 0.05, ^&⁣&^*P* < 0.01 vs. the G-Rb1 group, ns = statistical difference.

Cerebral blood flow (CBF) is closely related to nerve function. When CBF is significantly reduced, neurons will be irreversibly damaged. Early revasodilation after stroke is an important measure to save neurons in ischemic areas. CBF assessed by the laser speckle blood flowmeter in mice at 1, 3, 7, 14 days post-reperfusion (Figures 3A,B). The results showed that CBF in the IRI group was significantly reduced on 1th day, and then CBF was gradually restored with time compared with the Sham group, and finally recovered to 71% of baseline on 14th day. CBF in the G-Rb1 group was significantly increased at all-time points compared with the IRI group, especially at days 7 and 14. Compared with the G-Rb1 group, CBF in the G-Rb1 + XAV939 group was lower at all-time points. These results suggest that G-Rb1 could help restore CBF following MCAO/R, and the protective effect of G-Rb1 was canceled after adding XAV939. Furthermore, the protein expression level of vascular endothelial growth factor (VEGF) was measured on 7th and 14th day post-reperfusion to determine the effect of G-Rb1 on increasing blood vessel density (Figures 3C,D). VEGF expression in the G-Rb1 group was increased at days 7,14 compared with the IRI group (*P* < 0.01). Compared with the G-Rb1 group, VEGF expression was decreased after the addition of XAV939 (*P* < 0.05).

### G-Rb1 decreased microglial polarization to pro-inflammatory M1 type and promotes the polarization of microglia toward anti-inflammatory M2 type *in vivo*

**FIGURE 4:**
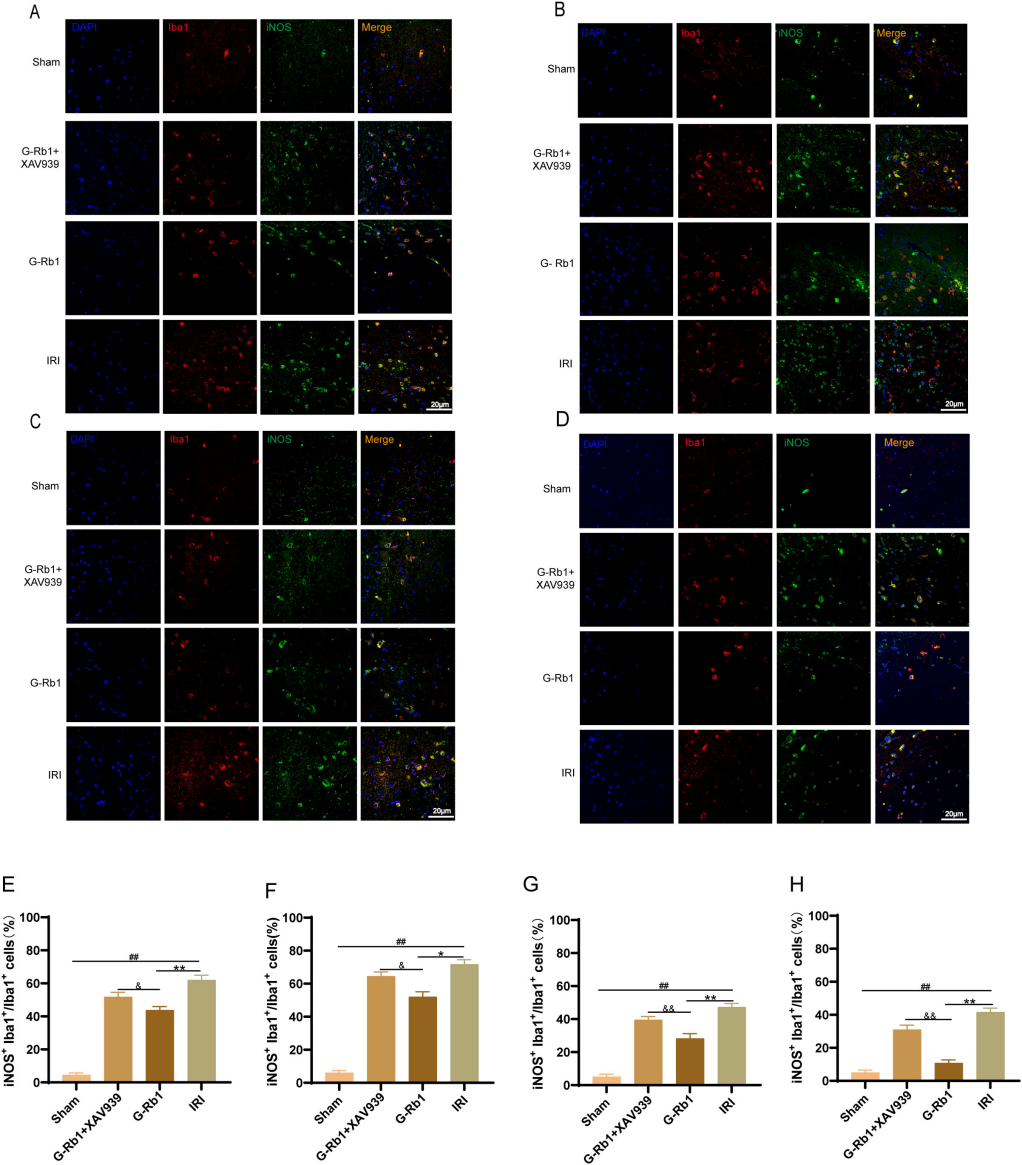
G-Rb1 decreased microglial polarization to pro-inflammatory M1 type. **(A–D)** Representative immunofluorescence images showing the expression of Iba1 and iNOS in microglia cells at 1, 3, 7, 14 days post-reperfusion of the mice (*n* = 3). **(E–H)** Statistical analysis of the proportion of Iba1^+^ iNOS^+^ cells at 1, 3, 7, 14 days post-reperfusion of the mice (*n* = 3 bar = 20μm). ^##^*P* < 0.01 vs. the Sham group, *P < 0.05, ^**^P < 0.01 vs. the IRI group, ^&^*P* < 0.05, ^&⁣&^*P* < 0.01 vs. the G-Rb1 group.

Immunofluorescence was used to detect the expression of microglia activated marker Iba1, M1 phenotypic maker iNOS, and M2 phenotypic maker Arg-1. These results showed that: compared with the Sham group, Iba1^+^ iNOS^+^ cells in the IRI group increased significantly at day 1,3,7,14 (Figure 4, *P* < 0.01), and the number of positive cells reached a peak at day 3. Compared with the IRI group, Iba1^+^ iNOS^+^ cells in the G-Rb1 group were significantly decreased at each time point (Figure 4, *P* < 0.05). Compared with the G-Rb1 group, Iba1^+^ iNOS^+^ cells in the G-Rb1 + XAV939 group increased at each time point (Figure 4, *P* < 0.05). The experimental results showed that the development of neuroinflammation after MCAO/R reached a peak at day 3, and then gradually recovered. G-Rb1 significantly inhibited microglia polarization toward pro-inflammatory M1 type at day 7 and 14, and the inhibitory effect was weakened after XAV939 was added.

**FIGURE 5:**
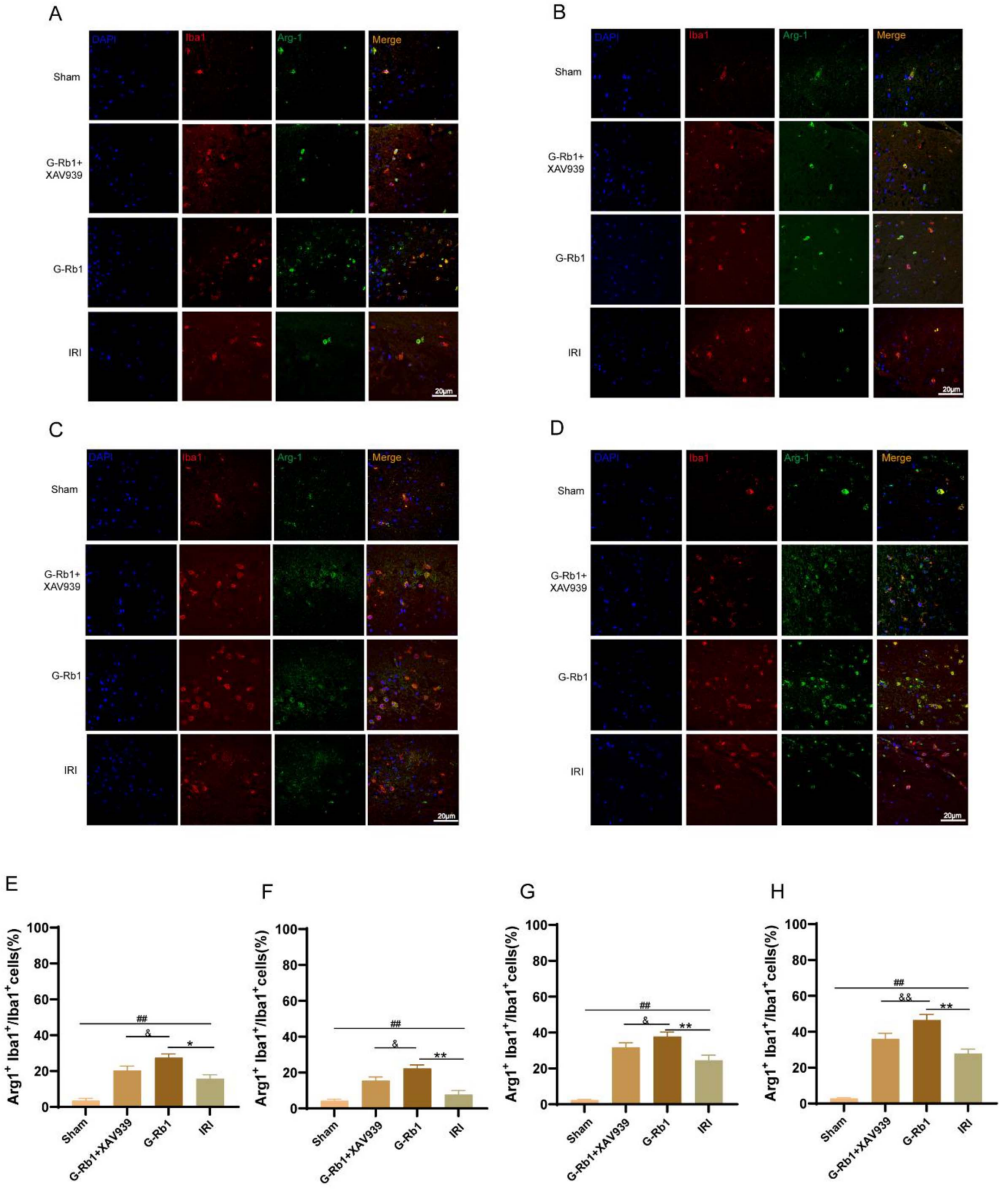
G-Rb1 increased microglial polarization to anti-inflammatory M2 type. **(A–D)** Representative immunofluorescence images showing the expression of Iba1 and Arg-1 in microglia cells at 1, 3, 7, 14 days post-reperfusion of the mice (*n* = 3). **(E–H)** Statistical analysis of the proportion of Iba1^+^ Arg-1^+^ cells at 1, 3, 7, 14 days post-reperfusion of the mice (*n* = 3 bar = 20 μm). ^##^*P* < 0.01 vs. the Sham group, *P < 0.05, ^**^P < 0.01 vs. the IRI group, ^&^*P* < 0.05, ^&⁣&^*P*<0.01 vs. the G-Rb1 group.

In addition, compared with the Sham group, Iba1^+^/Arg-1^+^ cells increased in the IRI group at 1, 3, 7, 14 days post-reperfusion (Figure 5, *P* < 0.01). Compared with the IRI group, Iba1^+^/Arg-1^+^ cells in the G-Rb1 group were significantly increased at each time point (*P* < 0.05). Compared with the G-Rb1 group, Iba1^+^/Arg-1 ^+^ cells in the G-Rb1 + XAV939 group were decreased at each time point (*P* < 0.05). The experimental results showed that following MCAO/R, microglia would polarize to M2 type in a small amount to counter the inflammatory response, while G-Rb1 increased the proportion of M2 type microglia at each time point, exerting anti-inflammatory effect. After addition of XAV939, the proportion of M2-type microglia decreased compared with the G-Rb1 group, demonstrating that the protective effect of G-Rb1 was attenuated by XAV939.

### G-Rb1 exerts anti-inflammatory effects in MCAO/R models via the Wnt/β-catenin signaling pathway

**FIGURE 6:**
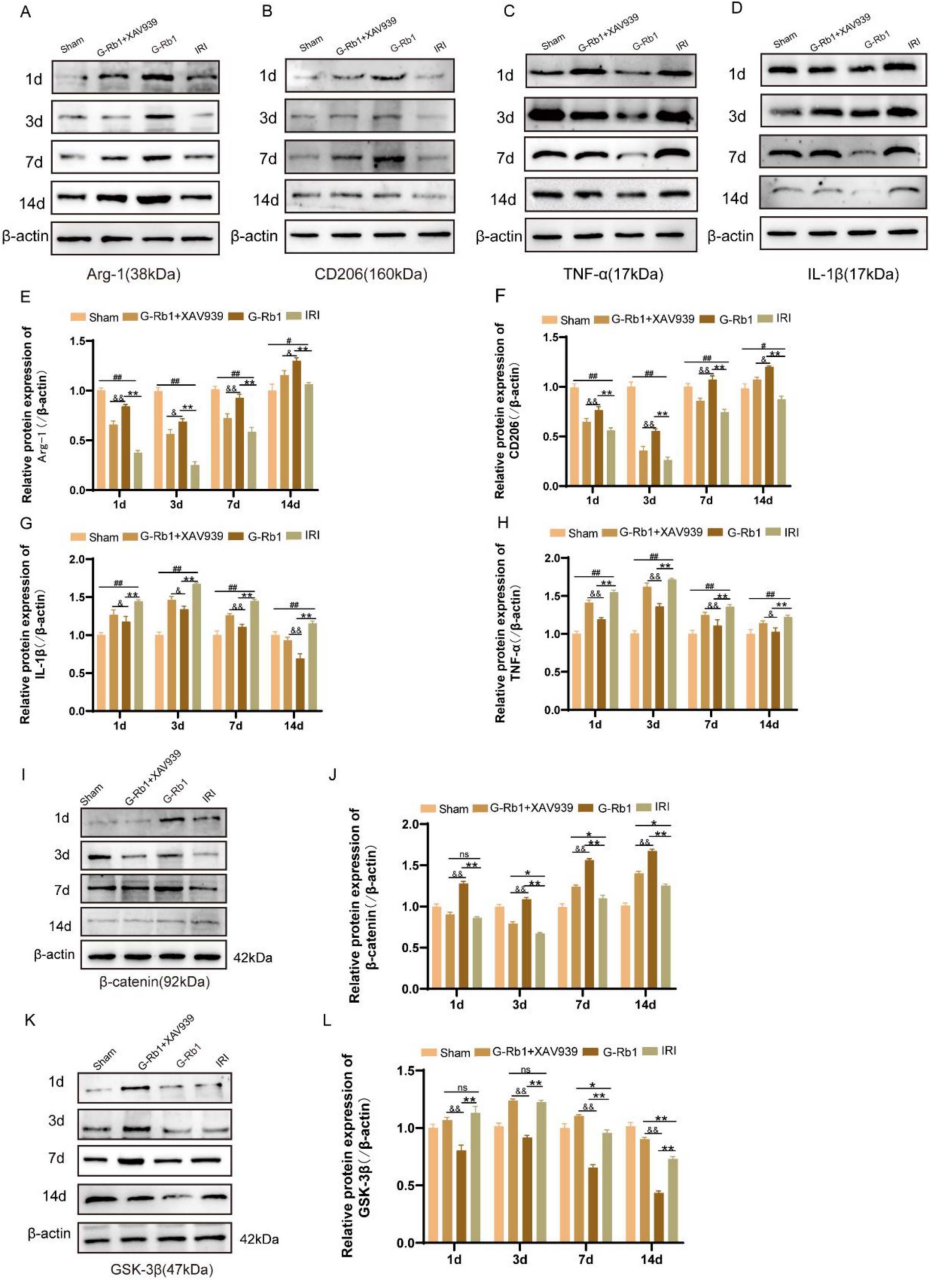
The protein expression of related inflammatory factors and related protein of the Wnt/β-catenin signaling pathway in each group at 1, 3, 7, 14 days post-reperfusion. **(A–H)** The protein expression of Arg-1 (A), CD206 (B), IL-1β **(C)** and TNF-α **(D)** at 1, 3, 7, 14 days post-reperfusion and Statistical analysis **(E–H)** (*n* = 3). **(I–L)** The protein expression of β-catenin **(I)**, GSK-3β **(K)** at 1, 3, 7, 14 days post-reperfusion and Statistical analysis **(J,L)** (*n* = 3). #*P* < 0.05, ##*P* < 0.01 vs. the Sham group, *P < 0.05, **P<0.01 vs. the IRI group, &*P* < 0.05, &&*P* < 0.01 vs. the G-Rb1 group, ns = statistical difference.

**FIGURE 7:**
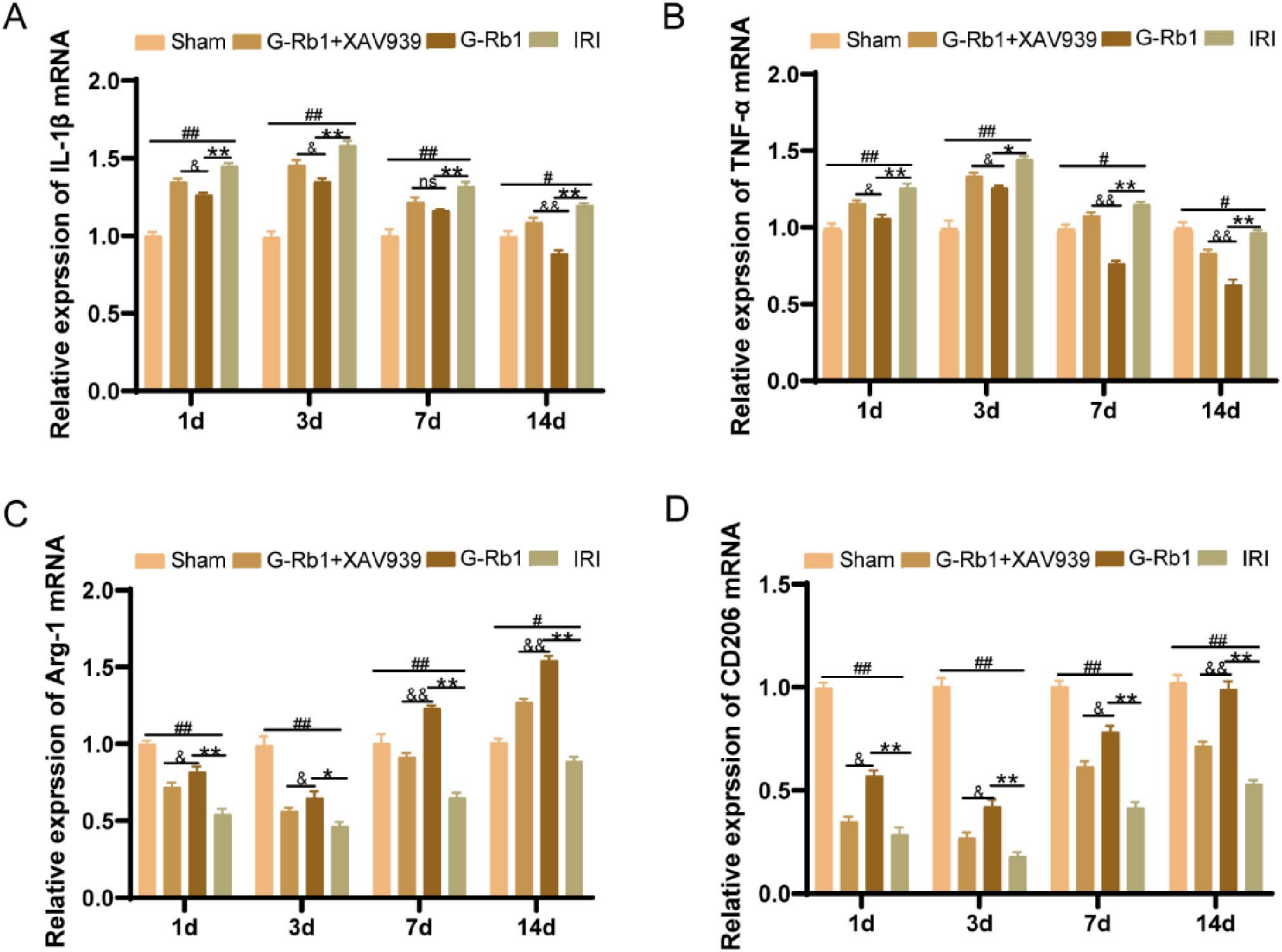
The mRNA levels of related inflammatory factors in each group at 1, 3, 7, 14 days post-reperfusion. IL-1β **(A)**, TNF-α **(B)**, Arg-1 **(C)**, CD206 **(D)** (*n* = 3). #*P* < 0.05, ##*P* < 0.01 vs. the Sham group, *P < 0.05, **P < 0.01 vs. the IRI group, &*P* < 0.05, &&*P* < 0.01 vs. the G-Rb1 group.

To further explore the role of G-Rb1-mediated activation of the Wnt/β-catenin signaling pathway during neuroinflammation, we detected changes in protein expression and mRNA levels of related inflammatory factors and key factors of Wnt/β-catenin signaling pathway in mice of different time points (Figures 6, 7). The results showed that the protein expressions of pro-inflammatory factors IL-1β and TNF-α in the IRI group were increased at 1, 3, 7, 14 days post-reperfusion (Figures 6A–H, *P* < 0.05), while the protein expressions of anti-inflammatory factors Arg-1 and CD206 were decreased at each time point compared with the Sham group (*P* < 0.05), and the changes of related inflammatory factor proteins were most significant on 3th day post-reperfusion. Compared with the IRI group, the protein expressions of pro-inflammatory factors IL-1β and TNF-α were decreased in the G-Rb1 group at days 1, 3, 7, and 14, while the protein expressions of anti-inflammatory factors Arg-1 and CD206 were increased, indicating that G-Rb1 can effectively reduce the expression of pro-inflammatory factors and increase the release of anti-inflammatory factors. And G-Rb1 showed a significant anti-inflammatory effect on 3th day of the most severe neuroinflammation (Figures 6A–H, *P* < 0.05). Compared with the G-Rb1 group, after blocking the Wnt/β-catenin signaling pathway with XAV939, the release of pro-inflammatory factors increased and the release of anti-inflammatory factors decreased in the G-Rb1 + XAV939 group at each time point, indicating that the protective effect of G-Rb1 was weakened. Changes in mRNA levels of related inflammatory factors were consistent with protein expression (Figures 7A–D).

To further investigate whether the neuroprotective effect of G-Rb1 is mediated by the Wnt/β-catenin signaling pathway, we examined the protein expression levels of GSK-3β and β-catenin at 1, 3, 7, 14 days post-reperfusion (Figures 6I–L). The results show: Compared with the IRI group, the protein expression of β-catenin in the G-Rb1 group was increased and the protein expression of GSK-3β was decreased at each time point (Figures 6J,L, *P* < 0.01), and β-catenin protein expression in the G-Rb1 + XAV939 group was not statistically different at day 1. The protein expression of GSK-3β showed no significant difference at days 1 and 3, but decreased at days 7, and 14 (Figure 6L, *P* < 0.05). Compared with the G-Rb1 group, β-catenin protein expression in the G-Rb1 + XAV939 group was significantly decreased at all-time points (Figure 6J, *P* < 0.05), while GSK-3β protein expression was increased (Figure 6L, *P* < 0.01).

## Discussion

Ginseng, as a traditional Chinese herbal medicine, has always been regarded for its therapeutic effects on central nervous system diseases (Radad et al., 2011; González-Burgos et al., 2015). Ginsenoside Rb1, one of the primary active components of ginseng, have many beneficial effects on vascular and central nervous system disease (Zhou et al., 2019). Investigating the neuroprotective effect of G-Rb1 on CIRI and its underlying molecular mechanism will provide further substantiation for clinical trials. Microglia are widely distributed throughout the cerebral cortex and play a crucial role in regulating neuronal activity. Therefore, we selected microglia within the cerebral cortex as our study subject. After brain injury, activated microglia do not exclusively exhibit a single phenotype but rather display characteristics of both M1 and M2 phenotypes (Hammond et al., 2019), as evidenced by the immunofluorescence results in BV2 cells. When the M1 phenotype predominates, microglia predominantly adopt a pro-inflammatory role, thereby exacerbating neuroinflammation. Conversely, when M2-type markers are more highly expressed, microglia exhibit anti-inflammatory properties. It is important to note that the M1/M2 paradigm represents a simplified model of microglial activation. Specifically, microglia are classified as M1-type when the M1 phenotype dominates, and as M2-type when the M2 phenotype is predominant. Following injury, the anti-inflammatory response diminishes rapidly, whereas the pro-inflammatory response persists for an extended period (Kumar et al., 2016). Thus, restoring and maintaining balance between the M1 and M2 phenotypes has emerged as a promising therapeutic strategy for mitigating neuroinflammation.

We verified the protective effect of G-Rb1 on CIRI through *in vivo* and *in vitro* models, and found that G-Rb1 played an important role in regulating microglia polarization. In addition, by adding XAV939, an inhibitor of Wnt/β-catenin signaling pathway, the regulatory function of Wnt/β-catenin signaling was clarified. G-Rb1 and/or XAV939 were intraperitoneally injected 3 days before the MCAO/R model. After modeling, neurobehavioral and cerebral blood perfusion were detected at 1, 3, 7, 14 days post-reperfusion. Cerebral infarction volume, microglia phenotype changes and expression of related inflammatory factors were detected after the mice were sacrificed. Before the OGD/R modeling, the BV2 cells were pre-treated with G-Rb1 and or XAV939 for 24 ho. And except the control group, LPS was added to the other groups to promote the polarization of BV2 microglia.

The experimental results showed that G-Rb1 has outstanding therapeutic effect in the following aspects: (1) G-Rb1 improved neurobehavioral, cerebral blood perfusion and brain infarction at 1, 3, 7, 14 days post-reperfusion; (2) G-Rb1 promoted the polarization of microglia from the M1 phenotype to the M2 phenotype *in vivo* and *in vitro* models; (3) G-Rb1 exerted neuroprotective effects by activating the Wnt/β-catenin signaling pathway, and XAV939 decreased the neuroprotective effect of G-Rb1.

Limited neurological recovery can lead to long-term impaired motor function after stroke. The Zea-Longa score and the mNSS neuroethological score were used to observe the behavior of mice to assess cortical neuron recovery. The score of the Zea-Longa and the mNSS were increased at all-time points of the IRI group compared with the Sham group and the neuroethological score of the G-Rb1 group was decreased at the same time compared with the IRI group. However, G-Rb1 and XAV939 co-treatment increased neurobehavioral scores and aggravated neurological deficits. The results showed that G-Rb1 has the therapeutic effect of improving nerve function deficit. We further examined the cerebral infarction volume and cerebral blood perfusion of mice at 1, 3, 7, 14 days post-reperfusion, and the results showed that the infarction volume of the G-Rb1 group decreased and cerebral blood perfusion recovery was obvious at each time point compared with the IRI group. However, co-treatment of XAV939 with G-Rb1 resulted in an increased volume of cerebral infarction and decreased cerebral blood perfusion when compared to the G-Rb1 group at each time point. In addition, the protein expression of vascular endothelial growth factor (VEGF) was measured on 7th and 14th days respectively, and we found that G-Rb1 can effectively promote angiogenesis after CIRI, while XAV939 co-treated with G-Rb1 can delay angiogenesis after cerebral ischemia. The result suggest that G-Rb1 can reduce infarct volume and promote angiogenesis by activating the Wnt/β-catenin signaling pathway.

Immunofluorescence was used to detect the expression of microglia activated maker Iba1, M1 phenotypic maker iNOS protein, and M2 phenotypic maker Arg-1 protein in the cerebral cortex. The IRI group showed severe neuroinflammatory reaction at 1, 3, 7, 14 days post- reperfusion compared with the Sham group, and the proportion of iNOS^+^ Iba1^+^ cells reached a peak on the 3th day and then decreased gradually, indicating that the inflammatory reaction was the most serious on 3th day after MCAO/R surgery. The proportion of Iba1^+^iNOS^+^ cells decreased and the proportion of Iba1^+^Arg-1^+^ cells increased in the G-Rb1 group at all-time points compared with the IRI group. After XAV939 co-treatment with G-Rb1, the proportion of Iba1^+^iNOS^+^ cells in parietal cortex of mice increased, while the proportion of Iba1^+^Arg-1^+^ cells decreased, which proved that XAV939 played an inhibitory role in the anti-inflammatory effect of G-Rb1.

The protein expression of related inflammatory factors was similar to that of quantitative (q) PCR and immunofluorescence. The expression of pro-inflammatory factors increased in the IRI group at all-time points, while the expression of anti-inflammatory factors decreased, and the expression of pro-inflammatory factors reached the highest on 3th day compared with the Sham group. However, G-Rb1 showed anti-inflammatory effects at all-time points. In addition, compared with the G-Rb1 group, the anti-inflammatory effects of G-Rb1 were canceled after XAV939 added, and the expression of pro-inflammatory factors was increased while the expression of anti-inflammatory factors was decreased at all-time points.

Immunofluorescence was used to detect the phenotypic polarization of BV2 cells. The results showed that BV2 cells exhibited an obvious M1 phenotype after the OGD/R model, characterized by increased the fluorescence intensity of iNOS, as compared with the Control group. Pretreatment with G-Rb1 weakened iNOS fluorescence intensity but enhanced Arg-1 fluorescence intensity compared with the LPS group. However, the co-treatment of G-Rb1 and XAV939 can make BV2 cells exhibit M1 phenotype, and Arg-1 fluorescence intensity is weakened while iNOS fluorescence intensity is increased. It demonstrated that pretreatment with G-Rb1 exerts anti-inflammatory benefits by increasing the number of M2-type microglia, while XAV939 co-treated with G-Rb1 counteracts this neuroprotective effect. Western Blot results showed that the expression of Arg-1 in the LPS group was decreased while the expression of iNOS was increased compared with the Control group, indicating that the release of pro-inflammatory factors and anti-inflammatory factors in BV2 cells was increased after co-treatment with ODG/R and LPS. The expression of anti-inflammatory factors increased and pro-inflammatory factors decreased in the G-Rb1 group compared with the LPS group, while the release of pro-inflammatory factors in BV2 cells was increased, and the release of anti-inflammatory factors was decreased compared with the G-Rb1 group after XAV939 was added. It suggested that the anti-inflammatory protective effect of G-Rb1 is mediated by activation of Wnt/β-catenin signaling pathway.

Angiogenesis and neuroplasticity are major factors in recovery from ischemic stroke, but they are often accompanied by persistent neuroinflammation. Recent studies have highlighted brain damage after CIRI aggravated by microglia-mediated neuroinflammation (Yin et al., 2018). It has been proved that inflammatory response after CIRI is mainly related to abnormal activation of microglia and infiltration of peripheral white blood cells (Carvalho et al., 2022). Activated microglia exhibit two types of M1 pro-inflammatory and M2 anti-inflammatory phenotypes, which are closely related to the occurrence and development of neuroinflammation. Therefore, regulation of activated microglia phenotypic polarization is an effective mechanism to control inflammatory response.

The Wnt signaling pathway is mainly involved in the occurrence and development of the central nervous system, and the Wnt/β-catenin signaling pathway is the most deeply studied among the Wnt pathways, and is highly conserved throughout the evolution process (Cailotto and Santulli, 2023). More and more evidence has proved that Wnt/β-catenin signaling pathway is closely related to the pathogenesis of CIRI (Jean LeBlanc et al., 2019; Jia et al., 2021). The Wnt/β-catenin signaling pathway is damaged in CIRI model, and activation of Wnt/β-catenin signaling pathway plays a neuroprotective role in ischemic stroke (Liu et al., 2022). In this study, experimental results confirmed that β-catenin expression increased and GSK-3β expression decreased after CIRI *in vivo*, demonstrating that Wnt/β-catenin signaling pathway was impaired in MCAO/R model, and β-catenin and GSK-3β expression were opposite in the G-Rb1 group. At the same time, the Wnt/β-catenin signaling pathway inhibitor XAV939 inhibited all neuroprotective effects of G-Rb1. Thus, the neuroprotective effect of G-Rb1 may be correlated with the activation of the Wnt/β-catenin signaling pathway.

Ginsenoside has been shown to maintain the integrity of the blood-brain barrier and reduce the incidence of hemorrhagic transformation in ischemic stroke (Hu et al., 2024), and its neurorepair effect may be related to the polarization of microglia from pro-inflammatory phenotype to anti-inflammatory phenotype mediated by β-catenin nuclear translocation. It has been proved that β-catenin can induce the polarization of proinflammatory and anti-inflammatory phenotypes and the release of related factors in monocyte derived dendritic cells (Azeem et al., 2020), and decrease the expression of proinflammatory factors while increase the expression of anti-inflammatory factors by increasing the nuclear content of β-catenin. These findings are consistent with previous experimental, demonstrating that β-catenin can promote the polarization of microglia toward anti-inflammatory phenotype and promote the recovery of inflammation. Experiments have shown that GSK-3β inhibitor TWS119 can effectively reduce the volume of cerebral infarction and improve neuroinflammation in the chronic phase of experimental stroke (Song et al., 2019). In this experiment, expression of GSK-3β of the G-Rb1 group decreased at all-time points, so G-Rb1 may improve neuroinflammation by reducing GSK-3β activity to activate Wnt/β-catenin signaling pathway. In addition, it is noteworthy that the changes in β-catenin protein levels in the ginsenoside Rb1 group were not positively correlated with the expression of anti-inflammatory factors across all time periods. This indicates that the neuroprotective effect of ginsenoside Rb1 on CIRI is not solely governed by a single pathway. Previous studies have demonstrated that ginsenoside Rb1 effectively reduces the level of phosphorylated NF-κB p65 in the MCAO/R model, thereby inhibiting the production of pro-inflammatory cytokines and preserving barrier integrity (Su et al., 2022). Moreover, inhibition of the PI3K/Akt signaling pathway has been shown to counteract the protective effects of ginsenoside Rb1 against neuronal death induced by transient ischemia (Luo et al., 2014). Therefore, whether the neuroprotective effects of ginsenoside Rb1 on ischemic stroke result from the combined actions of the Wnt/β-catenin signaling pathway and other inflammatory pathways merits further investigation.

Finally, the limitations of applying the mouse middle MCAO/R model to human stroke treatment must not be overlooked. First, interspecies differences in physiological and pathological mechanisms exist. Specifically, the proportion of white matter in the brain and the extent of collateral circulation in mice differ significantly from those in humans, potentially leading the MCAO/R model to underestimate the importance of white matter injury in stroke pathology. Second, the temporal dynamics and molecular pathways of the immune response to ischemic injury vary between mice and humans, which may contribute to the failure of certain anti-inflammatory targets to demonstrate efficacy in human clinical trials. Third, the mouse MCAO/R model is typically induced via mechanical obstruction, whereas human stroke etiology is multifactorial, involving complex vascular injury mechanisms. The suture method used in the MCAO/R model may not fully replicate the influence of thrombotic components on recanalization treatment. Additionally, experimental mice are generally young and healthy, contrasting sharply with clinical stroke patients, who are often older and have complications. Despite these limitations, it is undeniable that the mouse MCAO/R model remains the most effective disease model for simulating the key pathological processes of human stroke currently available. Moreover, the MCAO/R model offers distinct advantages, including experimental controllability, repeatability, and suitability for mechanistic research. It allows precise control over ischemia and reperfusion durations, fulfilling the research requirements for the “time window” in clinical thrombolytic therapy.

In summary, G-Rb1 showed a neuroprotective effect in both *in vivo* and *in vitro* models, which may be related to G-Rb1-mediated activation of Wnt/β-catenin signaling pathway. And it provides new insights into the pharmacological role of G-Rb1.

## Conclusion

G-Rb1 regulates the polarization of hypoxia-ischemia-activated microglia towards anti-inflammatory M2 phenotype by activating Wnt/β-catenin signaling pathway *in vivo* and *in vitro*, promoting the recovery of neuroinflammation and angiogenesis after CIRI, and ultimately improving CIRI.

## Data Availability

The original contributions presented in the study are publicly available. This data can be found in the following repositories: TTC staining: https://figshare.com/s/375f04de087a4aa8683c, Western blot: https://figshare.com/s/d1a7f7eb5bfc50eb2c00, Laser speckle flow: https://figshare.com/s/fbe606d90317c6284229.
